# Speed-resolved perfusion imaging using multi-exposure laser speckle contrast imaging and machine learning

**DOI:** 10.1117/1.JBO.28.3.036007

**Published:** 2023-03-20

**Authors:** Martin Hultman, Marcus Larsson, Tomas Strömberg, Ingemar Fredriksson

**Affiliations:** a Linköping University, Department of Biomedical Engineering, Linköping, Sweden; b Perimed AB, Stockholm, Sweden

**Keywords:** blood flow, microcirculation, multi-exposure laser speckle contrast imaging, artificial neural networks

## Abstract

**Significance:**

Laser speckle contrast imaging (LSCI) gives a relative measure of microcirculatory perfusion. However, due to the limited information in single-exposure LSCI, models are inaccurate for skin tissue due to complex effects from e.g. static and dynamic scatterers, multiple Doppler shifts, and the speed-distribution of blood. It has been demonstrated how to account for these effects in laser Doppler flowmetry (LDF) using inverse Monte Carlo (MC) algorithms. This allows for a speed-resolved perfusion measure in absolute units %RBC × mm/s, improving the physiological interpretation of the data. Until now, this has been limited to a single-point LDF technique but recent advances in multi-exposure LSCI (MELSCI) enable the analysis in an imaging modality.

**Aim:**

To present a method for speed-resolved perfusion imaging in absolute units %RBC × mm/s, computed from multi-exposure speckle contrast images.

**Approach:**

An artificial neural network (ANN) was trained on a large simulated dataset of multi-exposure contrast values and corresponding speed-resolved perfusion. The dataset was generated using MC simulations of photon transport in randomized skin models covering a wide range of physiologically relevant geometrical and optical tissue properties. The ANN was evaluated on in vivo data sets captured during an occlusion provocation.

**Results:**

Speed-resolved perfusion was estimated in the three speed intervals 0 to 1  mm/s, 1 to 10  mm/s, and >10  mm/s, with relative errors 9.8%, 12%, and 19%, respectively. The perfusion had a linear response to changes in both blood tissue fraction and blood flow speed and was less affected by tissue properties compared with single-exposure LSCI. The image quality was subjectively higher compared with LSCI, revealing previously unseen macro- and microvascular structures.

**Conclusions:**

The ANN, trained on modeled data, calculates speed-resolved perfusion in absolute units from multi-exposure speckle contrast. This method facilitates the physiological interpretation of measurements using MELSCI and may increase the clinical impact of the technique.

## 1 Introduction

Laser speckle contrast imaging (LSCI) is a noninvasive measurement technique for assessing spatial perfusion maps of the microcirculation.^1^ When coherent laser light scatters from moving red blood cells (RBCs) in tissue, it changes frequency due to the Doppler effect. Backscattered light from the tissue will therefore create a fluctuating speckle interference pattern on the camera sensor. These fluctuations cause motion blur in the recorded image, which can be quantified as the local contrast in a region of pixels. Several mathematical models have been proposed to establish a relation between the speckle contrast and blood flow speed or perfusion (speed × concentration). However, the amount of Doppler shifted light and the distribution of Doppler frequencies can affect a single-exposure contrast value in similar ways. This contributes to limit LSCI to relative estimates of perfusion in arbitrary units. It has been demonstrated that perfusion by LSCI follows a nonlinear and unknown relation to perfusion measured by laser Doppler flowmetry (LDF)^2^^–^^5^ and to actual tissue perfusion.^6^

Multi-exposure laser speckle contrast imaging (MELSCI) quantifies how the speckle contrast decreases with increasing exposure time, and allows for more realistic models of light–tissue interaction and speckle decorrelation when estimating perfusion.^7^ However, the mathematical models proposed for estimating perfusion from MELSCI are based on several assumptions,^8^ specifically that photons undergo at most one dynamic scattering event, and that the electric field autocorrelation function can be approximated as either a Gaussian or Lorentzian distribution.^9^ These assumptions are not valid for most tissues, as discussed in Refs. 10 and 11. Furthermore, using these models for analyzing MELSCI data yields relative perfusion estimates expressed in arbitrary units. This makes physiological interpretation difficult.

An alternative technique is to use a computational model based on Monte Carlo (MC) simulations in multilayered tissue models, where the limitations in the abovementioned assumptions are not necessary, and more complex light-tissue interactions can be considered. Specifically, in LDF it has been demonstrated how this, together with an inverse optimization algorithm, can be used to compute speed-resolved perfusion in absolute units %RBC × mm/s.^12^^–^^14^ By decomposing the perfusion into distinct speed components, each associated with a specific range of physiologically relevant speeds, more detailed and physiologically relevant information about the microcirculation is obtained. For example, this has been used to detect a reduced nutritive capillary flow in patients with type 2 diabetes.^15^

The drawback of inverse methods is the slow speed of the optimization when fitting the modeled data to measurements. Although this is viable for single-point LDF, it is too slow for an imaging modality such as MELSCI. To address this problem, machine learning has recently been proposed to train artificial neural networks (ANNs) that can estimate perfusion from multi-exposure contrast.^2^ Similar techniques have been used to estimate blood oxygen saturation from spectral data.^16^^,^^17^ This offloads the heavy computations to the training stage of the network and enables real-time estimations of microcirculation properties in imaging modalities.^18^

The aim of this study is to develop a method for speed-resolved perfusion imaging, computed from MELSCI data in the physiologically relevant unit %RBC × mm/s. The method utilizes ANN based on MC simulations of realistic complex tissue models accounting for expected variations in relevant skin properties. The result of the method is demonstrated in an arterial occlusion-release provocation.

## 2 Materials and Methods

### 2.1 Tissue Model

To generate realistic training data for the ANN, MC simulations of the light-tissue interaction in a three-layer skin tissue model were performed. This process has been described in detail elsewhere^6^^,^^16^ and will only be summarized here in the context of MELSCI. Optical properties used in the model are relevant for the laser wavelength 780 nm. The first layer in the tissue model is a bloodless epidermis layer with variable thickness ($t_{\mathrm{epi}}$) and melanin concentration. The absorption coefficient of the epidermis layer ($\mu_{a,\mathrm{epi}}$) is dependent on melanin and is generally low at the used laser wavelength. The second and third layers are dermis layers with variable blood tissue fractions, blood speed distributions, scattering coefficients, and vessel diameters. The upper dermis layer has a fixed thickness of 0.2 mm, and the lower dermis layer is infinite. The reduced scattering coefficient ($\mu_{s}^{\prime }$) is the same in all layers. The absorption coefficient of the dermis layers ($\mu_{a,\text{dermis}}$) is given by their blood tissue fraction ($c_{\text{blood}}$), oxygen saturations ($S_{\mathrm{oxy}}$), and vessel diameters ($d_{\text{vessels}}$), where the latter affects the absorption through the vessel packaging effect.^19^^,^^20^ The parameters $c_{\text{blood}}$, $S_{\mathrm{oxy}}$, and $d_{\text{vessels}}$ are modeled to be different in the two dermis layers, where the actual values are calculated from a randomized average value and a randomized difference parameter, to ensure the parameters do not differ too much between the layers.

**Fig. 1:**
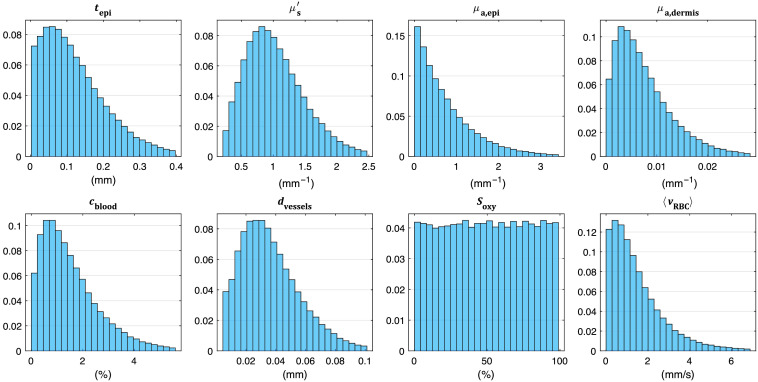
Normalized histograms of skin parameter distributions in the tissue models. The x-axes are scaled to remove the 1% upper tail.

The parameters of interest in the 100,000 randomized training models are summarized in Fig. 1. The average values for $c_{\text{blood}}$, $S_{\mathrm{oxy}}$, and $d_{\text{vessels}}$ are given in the histograms. The last histogram shows the average speed $\left\langle v_{\mathrm{RBC}}\right\rangle$ of the RBC speed distributions, described in more detail below. The choice of the distributions was based on three principles; (1) the range of the distributions should cover essentially all expected values in skin tissue for all skin sites and all skin types; (2) allow for less probability of parameter values that are rarely expected, such as $\mu_{s}^{\prime }<0.5\;{\mathrm{mm}}^{-1}$^21^; (3) have higher probability for ranges where a small change has a large effect on the contrast data, thus exponential decay in the histograms of many of the parameters as Beer–Lambert’s law predicts lower absolute change for high $\mu_{a}$ or large path-lengths (related for example to high $t_{\mathrm{epi}}$).

**Fig. 2:**
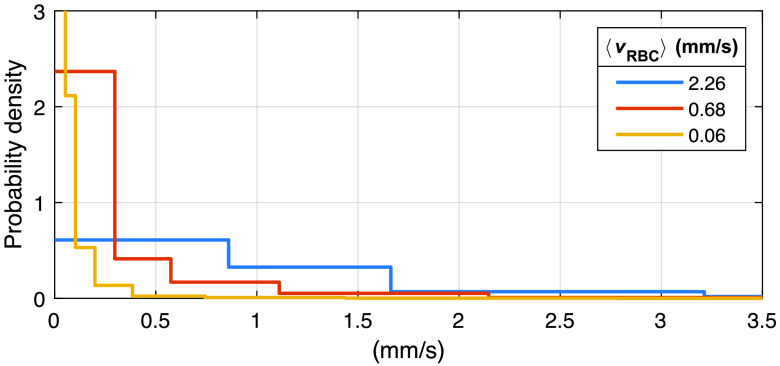
Examples of RBC speed distributions $c_{\mathrm{RBC}}(v)$, for three different mean speeds as presented in the legend. The distributions were normalized to have a total probability of 1.

When modeling speckle contrast, it is common to assume either a Gaussian or Lorentzian distribution for the electric field autocorrelation function,^9^^,^^22^ but the validity of both assumptions has been questioned.^11^ Here, we instead model the speed distribution of RBCs, $c_{\mathrm{RBC}}(v)$, directly as a weighted sum of 10 uniform distributions from 0  mm/s to exponentially increasing top speeds, representing vessels with parabolic flow profiles with 10 different average speeds. The weights are randomized for each tissue model to create variations in the shape of the distribution. Examples of speed distributions are presented in Fig. 2. Additional details can be found in Ref. 6.

### 2.2 Doppler Histogram Calculation

Tissue model simulations were performed for calculating Doppler histograms (the optical Doppler spectrum) as previously described in detail.^13^ The simulations and calculations are summarized in this section.

MC simulations were performed with varying epidermis thickness and tissue scattering covering the ranges found in Fig. 1 for these parameters. In each MC simulation, the total path length of each photon in each of the three layers was stored and used to calculate path-length distributions in each layer. For a given set of the model parameters $t_{\mathrm{epi}}$ and $\mu_{s}^{\prime }$, the path-length distribution in each layer was interpolated from the simulated path-length distributions. Using the path-length distribution in each layer, Beer–Lambert’s law was applied to add the absorption effect of $\mu_{a,\mathrm{epi}}$ and $\mu_{a,\text{dermis}}$, modifying the path-length distributions. These modified path-length distributions were the first input to the calculation of the Doppler histogram of the model.

A single-shifted Doppler histogram was calculated for light Doppler shifted once by a red blood cell with a certain speed. This was done analytically as described in the appendix of Ref. 13, assuming a random angle between the light and the direction of the red blood cell and a scattering angle based on the Gegenbauer kernel phase function.^23^ The phase function parameters were set to $g_{\mathrm{Gk}}=0.948$ and $\alpha_{\mathrm{Gk}}=1.0$, resulting in an anisotropy factor of 0.991.^24^ The Doppler histogram for light shifted n times was calculated as the cross-correlation of the single-shifted Doppler histogram with itself n−1 times. Probability distributions of the number of Doppler shifts were given by Poisson distributions based on the pathlengths and fraction of red blood cells. The fact that blood is confined in distinct blood vessels rather than being evenly distributed in the layer, i.e. the vessel packaging effect, was also accounted for when calculating the distribution of the number of Doppler shifts.

Finally, the speed distribution, the path-length distributions, the distributions of the amount of Doppler shifts, and the Doppler histograms for various numbers of Doppler shifts and speeds, were used to calculate a final Doppler histogram H(f). Although the entire process is complex, the calculation scheme is efficient, and it takes less than a millisecond to calculate a Doppler histogram for a specific set of model parameters.

### 2.3 Speckle Contrast Calculation

The Doppler histogram H(f) obtained as described in the previous section was converted into multi-exposure contrast K(T) using a mathematical framework that is thoroughly described in Refs. 6 and 25. In short, the electric field autocorrelation function $g^{\left(1\right)}(\tau )$ was obtained from the Doppler histogram using the Wiener–Khinchine theorem

$$g^{\left(1\right)}(\tau )=|F^{-1}\{H(f)\}|,$$ (1)

where $F^{-1}$ is the inverse Fourier transform. The contrast was then obtained as^26^

$$K^{2}(T)=\frac{2\beta }{T}\int_{0}^{T}{\left|g^{\left(1\right)}(\tau )\right|}^{2}(1-\frac{\tau }{T})d\tau ,$$ (2)

where the coherence factor β was set to one, assuming a calibrated system with K=1 for a static target.

The contrast computed by this method is noise-free, making it inappropriate for training models that should be applied to real data containing noise. Therefore, a noise component $K_{\text{noise}}(T)$ was added to the simulated contrast data before training the neural network. The noise component model has been described and validated in Ref. 2. In summary, speckle simulations showed that the noise for each exposure time T could be modeled as a sequence of random contrast offsets depending on the average contrast over all exposure times ${\left\langle K(T)\right\rangle }_{T}$ and the noise value at the previous exposure time, i.e.,

$$K_{\text{noise}}(T)={\left\langle K(T)\right\rangle }_{T}\times \{\begin{array}{ll} \xi_{T}\eta_{1}, & T=1\\ K_{\text{noise}}(\frac{T}{2})+\xi_{T}\eta_{\mathrm{diff}}(T), & T=[2,4,8,16,32,64] \end{array},$$ (3)

where $\xi_{T}$ are random numbers drawn from a normal distribution with zero mean and unit variance, and the constants $\eta_{1}$ and $\eta_{\mathrm{diff}}(T)$ were determined from the speckle simulations and dependent on the number of speckles per detector pixel.

### 2.4 Biological Zero

When using the laser Doppler technique in measurements at low-flow or zero-flow conditions in skin tissue, for example during brachial occlusion, we have observed a residual high frequency energy in the Doppler power spectrum (ongoing work), corresponding to the well-known biological zero (BZ) effect.^27^^,^^28^ During occlusion, BZ partially originates from Brownian motion of RBCs and from the redistribution of blood between vessels, possibly including reverse flow in the capillaries.^29^ In addition, movements of macromolecules in the interstitial space contribute to the BZ.^30^ Due to the small size of these macromolecules, the scattering angles are large compared to scattering from RBCs, resulting in large Doppler shifts. This addition to the power spectrum cannot be explained by Doppler shifts from RBCs, regardless of which speed distributions of the RBCs are used. Furthermore, the residual energy is not present when measuring on flow of phantoms perfused with whole blood,^14^ making us confident in assuming that it does not originate from the blood itself, but from some kind of other tissue movements, well in line with the movement of macromolecules. We observe the same effect when using the MELSCI technique. Similar to the residual we observe in the Doppler spectrum, the shape of the contrast curve in measured data during occlusion cannot be explained by any realistic speed distribution of the RBCs. However, since we observe the same phenomenon using both LDF and MELSCI, we are confident that it does not originate from faulty calibrations or instrument noise.

We must account for this macromolecule effect on BZ when generating training data from the skin models, otherwise, the training data will not be valid for low-flow conditions. This can be done by adding a BZ component $H_{\mathrm{BZ}}$ to the Doppler histogram before computing the contrast. This was empirically modeled as

$$H_{\mathrm{BZ}}(f)=\{\begin{array}{ll} 0 & \text{for }f=0\\ 10^{\xi_{\text{offset}}+f\xi_{\text{slope}}} & \text{for }f>0 \end{array},$$ (4)

where $\xi_{\text{offset}}\in N(-5.3,0.125)$ and $\xi_{\text{slope}}\in N(-5.2\times 10^{-5},6\times 10^{-6})$ represent random numbers from normal distributions N(μ,σ). These distributions are based on analysis of laser Doppler spectra recorded during occlusion provocations in a substudy of the Swedish cardiopulmonary bioimaging study (SCAPIS).^31^ Note that $H_{\mathrm{BZ}}$ has a negligible additive effect on the Doppler histogram under normal flow conditions.

### 2.5 Perfusion

Speed-resolved perfusion can be computed from the modeled speed distribution as

$$P_{\left[v_{\min},v_{\max}\right]}=\int_{v_{\min}}^{v_{\max}}vc_{\mathrm{RBC}}(v)dv,$$ (5)

where the perfusion corresponds to the speed-range $\left[v_{\min},v_{\max}\right]$, and $c_{\mathrm{RBC}}(v)$ is the speed distribution describing the concentration of RBCs moving with speed v. This distribution sums to the total tissue RBC fraction and has the unit %RBC/(mm/s). The unit of speed-resolved perfusion is thus %RBC × mm/s, i.e. concentration × speed. In this study, we present speed-resolved perfusion in the ranges 0 to 1  mm/s, 1 to 10  mm/s, and >10  mm/s. Additionally, the total perfusion, i.e. the sum of all three speed components, is presented.

When calculating the modeled perfusion $P_{\text{true}}$, the contribution from each skin layer is considered so that $P_{\text{true}}$ reflects the sampling volume. For example, if the epidermis thickness is increased but blood tissue fractions and speed distributions are kept constant in the dermis layers, the total perfusion will slightly decrease since the epidermis layer (with zero perfusion) will constitute a larger part of the sampling volume. See Ref. 13 for further details.

For comparison, we also compute a perfusion estimate from single-exposure contrast as

$$P_{\mathrm{SE}}(T)=\frac{1}{K(T)}-1.$$ (6)

There are other single exposure models where P is proportional to $K^{-2}$, e.g. Ref. 32. We have done the analysis using this model as well, but the performance was quantitatively worse than for Eq. (6), and the conclusions were unchanged by this choice. Furthermore, Eq. (6) is relatively common in clinical settings as it is used in the PeriCam PSI (Perimed AB, Järfälla, Stockholm, Sweden), and thus a good comparison for the potential benefits of our new model.

### 2.6 Artificial Neural Network Perfusion Model

A neural network was trained on the data from the tissue model to estimate speed-resolved perfusion in a single pixel of the multi-exposure contrast images. Thus, the network had seven inputs representing squared multi-exposure contrast $K^{2}(T)$ calculated at T=[1,2,4,8,16,32,64] ms exposure time and three outputs representing speed-resolved perfusion in the ranges 0 to 1, 1 to 10, and >10  mm/s. The neural network was a fully connected network with a single hidden layer with 25 nodes and hyperbolic tangent (tanh) activation. Linear activation was used for the output layer. To obtain a perfusion image, the model was applied to each individual pixel of the contrast image. The architecture was determined by the process described in Sec. 2.6.1. The dataset consisted of 100,000 tissue models, divided into 70/15/15% for training, validation, and test, respectively. The validation set was used for early stopping and in-training hyperparameter tuning, and the test set was used to get an unbiased performance metric for comparison of multiple networks. 25 networks were trained from random initializations, and the best performing on the 15% test-split was selected as the final model. This model was then evaluated on a separate dataset of 100,000 tissue models, to get the final unbiased performance metrics. The training was done using the Deep Learning toolbox in MATLAB 2021b (MathWorks Inc., MA).

The model was trained using a weighted mean square error (wMSE), where the individual errors of each speed-component were weighted by their contribution to the total perfusion. Specifically,

$$\mathrm{wMSE}(j)=\frac{1}{N}\overset{N}{\underset{i=1}{\sum }}\frac{(P_{\mathrm{ANN},j,i}-P_{\text{true},j,i}{)}^{2}}{P_{\text{true},\mathrm{tot},i}},\;j=\{1,2,3\},$$ (7)

where

$$P_{\text{true},\mathrm{tot},i}=\overset{3}{\underset{j=1}{\sum }}P_{\text{true},j,i},$$ (8)

and j indicates the speed component (0 to 1, 1 to 10, >10  mm/s, respectively, for j=1, 2, and 3), $P_{\mathrm{ANN}}$ is the network output (predicted speed-resolved perfusion), $P_{\text{true}}$ is the target output (true speed-resolved perfusion calculated directly from the tissue model), and N is the number of samples, i.e. number of models in the training, validation, or test set. For evaluation, the weighted mean absolute percentage error (wMAPE) was used, due to the easier interpretability

$$\mathrm{wMAPE}(j)=\frac{100}{N}\overset{N}{\underset{i=1}{\sum }}\frac{\left|P_{\mathrm{ANN},j,i}-P_{\text{true},j,i}\right|}{P_{\text{true},\mathrm{tot},i}},\;j=\{1,2,3\}.$$ (9)

For each speed component j, this metric informs the prediction error relative to the total perfusion. This accounts for the speed distribution and does not inflate small relative errors at low perfusion values, unless the total perfusion is also low. An example is warranted.

Suppose we have the true speed-resolved perfusion $P_{\text{true}}=[0.01,0.1,1]$, and the prediction $P_{\mathrm{ANN}}=P_{\text{true}}+0.01=[0.02,0.11,1.01]$. The unweighted absolute percentage error would be $\mathrm{MAPE}=100|(P_{\mathrm{ANN},j}-P_{\text{true},j})/P_{\text{true},j}|=[100,10,1]\%$, whereas the weighted absolute percentage error would be $\mathrm{wMAPE}=100|(P_{\mathrm{ANN},j}-P_{\text{true},j})/P_{\text{true},\mathrm{tot}}|\approx [0.9,0.9,0.9]\%$, better reflecting the fact that the low speed-component is small in this case and thus should not be given as much weight in the total error.

Empirical results confirmed that using wMSE for training resulted in better overall performance compared to unweighted MSE. Note that wMAPE is only an applicable metric for the speed components. For total perfusion and single-exposure perfusion, unweighted MAPE is used instead.

We also present the coefficient of determination, $R^{2}$, between estimated and true perfusion, which represents the proportion of the variance in perfusion explained by the model.

#### 2.6.1 Choice of ANN architecture

To determine the optimal ANN architecture described above, considering both accuracy and computational complexity, a large number of network architectures were evaluated in a hyperparameter grid search over number of hidden layers and their sizes, activation functions, and regularization weights. The results showed that two hidden layers performed slightly better than the single hidden layer architecture but not enough to warrant the increase in computations. Similarly, performance increased with larger layers but did not warrant a size above 25 nodes. The small size also enables efficient real-time implementations. Using $L_{2}$-regularization did not significantly affect the results and thus was not used in the final model. This is likely due to the small size of the model, making overfitting unlikely. Finally, the activation functions tanh [Eq. 10(a)], rectified linear unit [ReLU, Eq. 10(b)], saturated linear unit [SatLU, Eq. 10(c)], and softplus [Eq. 10(d)] were investigated. Tanh performed best and softplus only slightly worse, whereas ReLU and SatLU performed significantly worse, possibly due to the nonsmooth transitions at 0 for ReLU and at 0 and 1 for SatLU

$$\tanh(x)=(e^{2x}-1)/(e^{2x}+1),$$ (10a)

ReLU(x)=max(0,x), (10b)

SatLU(x)=min(1,max(0,x)), (10c)

$$\text{Softplus}(x)=\log(1+e^{x}).$$ (10d)

To enable the use of the model in clinical applications, real-time processing is a necessity. For this reason, the models were kept as small as possible without impacting accuracy. The average processing time for one 180×320×7 multi-exposure contrast image was 33 ms using a single-threaded MATLAB implementation running on a 3.5 GHz Intel Xeon CPU. Despite not being the focus of this study, this model already runs in real-time. More optimized implementations would likely reduce this time further.

#### 2.6.2 Restricted tissue model

In addition to the main ANN model, we trained a separate model on a restricted training set, where all tissue parameters not directly related to perfusion were set to the median of their respective distributions in Fig. 1. In other words, $t_{\mathrm{epi}}$, $\mu_{s}^{\prime }$, $\mu_{a,\mathrm{epi}}$, $d_{\text{vessels}}$, and $S_{\mathrm{oxy}}$ were fixed at one value in all 100,000 tissue models, whereas $c_{\text{blood}}$ and the parameters of the speed distribution were unchanged from the original distributions. Note that $\mu_{a,\text{dermis}}$ still varied due to changes in $c_{\text{blood}}$. Additionally, the noise component $K_{\text{noise}}(T)$ was omitted from this data. This dataset represents a situation where the only influence on the speckle contrast is from the parameters directly determining the perfusion. This allows investigating the core method without confounding factors and sets a rough upper bound on the achievable performance. The network architecture of this model was the same as that of the main model.

### 2.7 *In-Vivo* Measurements and Measurement System

The MELSCI instrument used in this study has been thoroughly described in previous publications.^18^^,^^33^ In summary, the technique is based on the continuous acquisition of images with 1 ms exposure time from a 1000 frames-per-second high-speed CMOS sensor. The images are successively accumulated to create longer synthetic exposure times at 2, 4, 8, 16, 32, and 64 ms.^33^ The use of synthetic exposure times was first proposed and validated in Ref. 34. Further, the accumulation and speckle contrast algorithms are implemented in a field-programmable gate array (FPGA) closely integrated with the CMOS sensor. This enables continuous real-time acquisition and processing of multi-exposure contrast images, without any loss of information due to the negligible interframe time.^18^ Each set of 64 speckle images results in one multi-exposure contrast image, enabling a framerate for perfusion images of 15.6 frames-per-second.

An arterial occlusion-release provocation of the forearm was performed in a healthy 24-year-old male volunteer. The subject did not consume any caffeine or nicotine for 6 h prior to the experiment and rested for 15 min before the measurement in a 23°C temperature-controlled room. The protocol followed 5+5+5  min of baseline, brachial occlusion at 200 mmHg, and reperfusion, and was approved by the Regional Ethical Review Board in Linköping, Sweden (D.no. 2018/282-31). Written informed consent was given by the volunteer before the experiment.

## 3 Results

### 3.1 ANN Simulation Performance

**Fig. 3:**
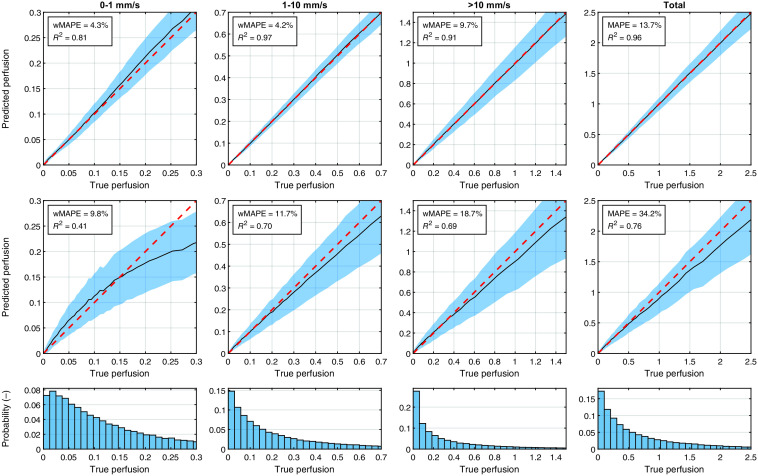
The top row shows perfusion predicted by the ANN trained on the restricted tissue model compared to true perfusion [%RBC × mm/s]. The second row shows the same for the main model. The data were divided into 50 bins, each with 2000 data points, based on the true perfusion (x-axis). The black lines are the mean predicted perfusion in the bins, and the blue areas indicate the average deviation from the mean in each bin. The red dashed line is the ideal theoretical prediction. Summary metrics weighted mean absolute percentage error (wMAPE) for speed-resolved perfusion, mean absolute percentage error (MAPE) for total perfusion, as well as $R^{2}$, is presented for both models. The histograms show the distribution of true perfusion in the dataset.

The performance of the main and restricted ANN models on the simulated evaluation sets is presented in Fig. 3. The three speed components were evaluated using the wMAPE metric [Eq. (9)] and total perfusion was evaluated using MAPE. The coefficient of determination $R^{2}$ between predicted and target perfusion was also computed for all four perfusion estimates. The data was divided into 50 bins with 2000 points each, according to the true perfusion (x-axis). The mean predicted perfusion in each bin is shown by the black line, with the blue area representing the average deviation from the mean. The ideal prediction is indicated by the red dotted line. The top row shows the performance of the restricted model with only perfusion parameter variation and no noise, and the second row is the main model. The histograms in the last row show the distribution of true perfusion in the dataset.

The second row of Fig. 3 demonstrates the combined effect on perfusion from all skin parameters in many randomized models. The relatively low $R^{2}$ for the lowest speed component for the main model ($R^{2}=0.41$; 0 to 1  mm/s), increased with higher speed and for total perfusion. The same is true for the restricted model presented in the first row, where all metrics are significantly better than the main model case.

**Fig. 4:**
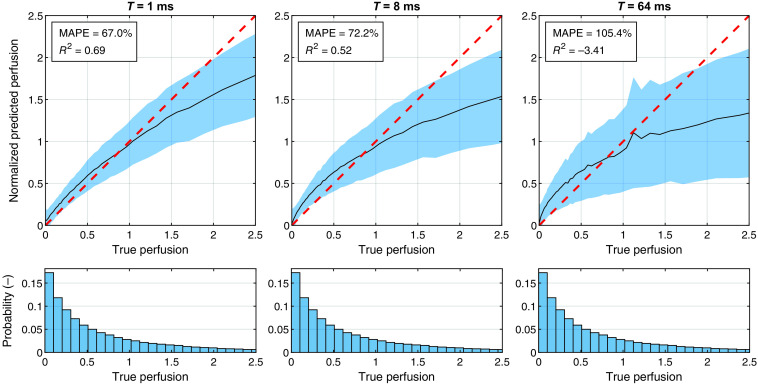
Predicted single-exposure perfusion $P_{\mathrm{SE}}(T)$ [PU] evaluated against true total perfusion (%RBC × mm/s). To allow comparison between the arbitrary perfusion units and absolute perfusion units, the predicted values were normalized to have the same average as true perfusion in the displayed range. As in Fig. 3, the histograms show the distribution of true perfusion in the dataset.

To put these values in context, Fig. 4 shows similar performance plots as Fig. 3, using the main model for generating evaluation data, but for single-exposure perfusion estimated with Eq. (6). To allow comparison between the arbitrary perfusion units from the single-exposure model and the absolute units of true perfusion, the estimated perfusion was normalized to have the same mean as true perfusion in the range 0 to 2.5%RBC × mm/s. This normalization was not done for the ANN model but would only have had a small effect on the current values. Regardless, the single-exposure model performs worse than the ANN model for both metrics (MAPE and $R^{2}$) at all exposure times. Note that the plots in Fig. 4 compare with total perfusion and thus should only be compared to the fourth column in Fig. 3.

**Fig. 5:**
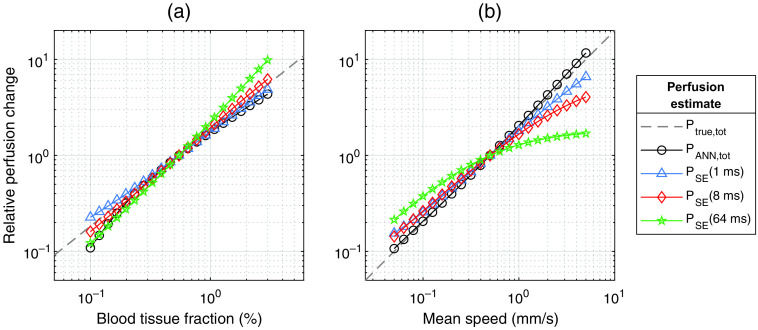
Relative change in perfusion estimates due to a change in (a) blood tissue fraction and (b) mean blood speed. Changes are relative to perfusion estimates at (a) 0.55% and (b) 1 mm/s, respectively. Each point is the median change in 5000 tissue models randomly selected from the test dataset. An ideal method should follow the dashed line. Note the log-log scales.

**Fig. 6:**
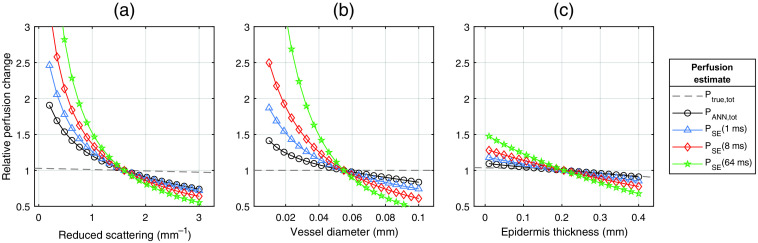
Relative change in perfusion estimates due to a change in (a) reduced scattering coefficient, (b) vessel diameter, and (c) epidermis thickness, indicating the largest sources of error in the absolute value of the estimated perfusion. Changes are relative to (a) $1.6\;{\mathrm{mm}}^{-1}$, (b) 0.055 mm, and (c) 0.205 mm, respectively. Each point is the median change in 5000 tissue models randomly selected from the test dataset. An ideal method should follow the dashed line.

To further evaluate how specific skin parameters influence the perfusion, one skin parameter at a time was changed over a wide range of values while the rest of the parameters were unchanged, as presented in Figs. 5 and 6. The relative change in four perfusion estimates, $P_{\mathrm{ANN},\mathrm{tot}}$, $P_{\mathrm{SE}}(1\;\mathrm{ms})$, $P_{\mathrm{SE}}(8\;\mathrm{ms})$, and $P_{\mathrm{SE}}(64\;\mathrm{ms})$ was compared to the relative change in true perfusion $P_{\text{true},\mathrm{tot}}$. Fig. 4(a) shows the median relative change in perfusion in 5000 tissue models due to changes in blood tissue fraction. Both $P_{\mathrm{ANN},\mathrm{tot}}$ and $P_{\mathrm{SE}}(8\;\mathrm{ms})$ had close to linear responses. Figure 5(b) shows similar changes when varying the mean blood speed. Here, $P_{\mathrm{ANN},\mathrm{tot}}$ had a significantly more linear response compared to all single-exposure perfusion estimates.

Figure 6 shows the median relative change in perfusion due to changes in reduced scattering coefficient, vessel diameter, and epidermis thickness, where almost no change is expected in the ideal case (some minor changes due to changes in sample volume are expected in the ideal case). $P_{\mathrm{ANN},\mathrm{tot}}$ was highly sensitive to changes in scattering coefficient [Fig. 6(a)], although less sensitive than the single-exposure methods. While $P_{\mathrm{ANN},\mathrm{tot}}$ was less sensitive to vessel diameter than the single-exposure methods, it still deviated by up to 40% for very small vessel diameters [Fig. 6(b)]. $P_{\mathrm{ANN},\mathrm{tot}}$ was almost insensitive to changes in the epidermis thickness, whereas the single-exposure methods were increasingly sensitive with higher exposure time.

### 3.2 *In-Vivo* Measurements

**Fig. 7:**
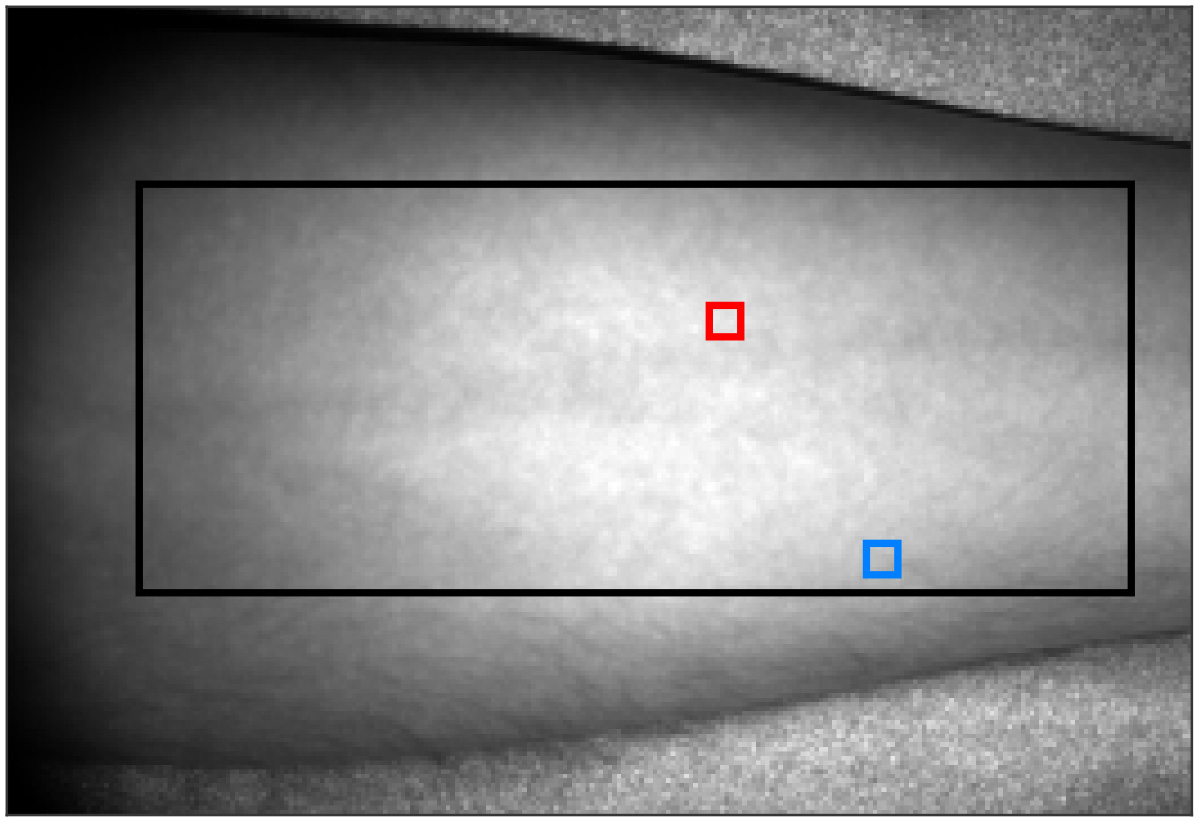
Intensity image of the forearm presented in the occlusion-release experiment. The large black ROI was used to automatically determine the color scale for the perfusion images in Figs. 8 and 9, and for studying spatial variations in the perfusion. The red and blue ROIs were used to extract the time traces in Figs. 10 and 11 in a high-flow and low-flow region, respectively.

**Fig. 8:**
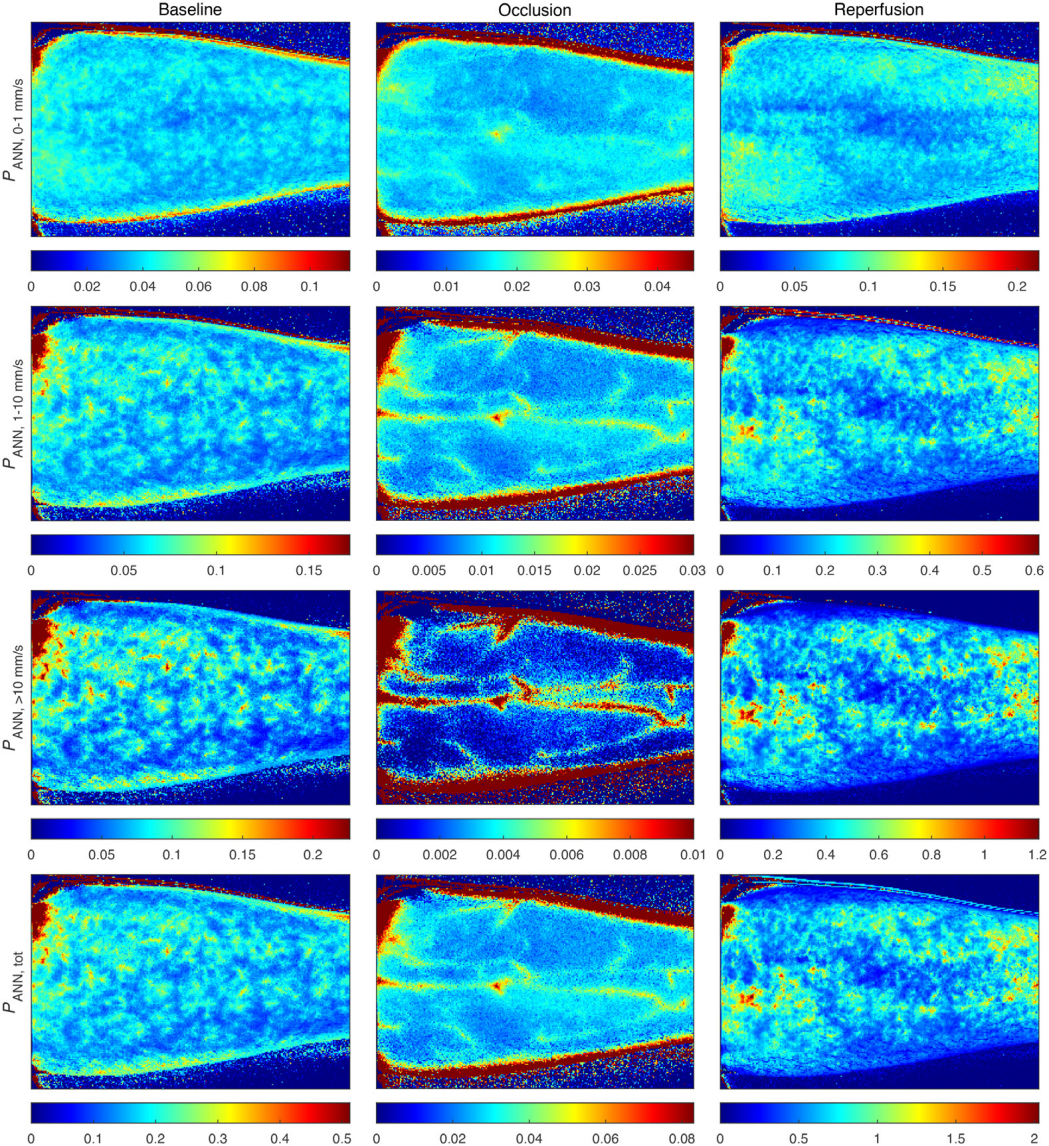
*In-vivo* measurement of a forearm during an occlusion-release provocation. Columns represent the three phases of the provocation; baseline, occlusion, and reperfusion. Rows represent the three speed components and total perfusion. The color scale in each image was selected for all images to be visually comparable, using three times the mean perfusion in the large ROI in Fig. 7. Perfusion scales in the unit %RBC × mm/s are displayed in the colorbar below each image. The high perfusion area in the upper left corner of the images is an artifact due to a low intensity. Similarly, the visually high perfusion at the edges of the arm in the occlusion images is due to the low perfusion scale in combination with the low intensity.

**Fig. 9:**
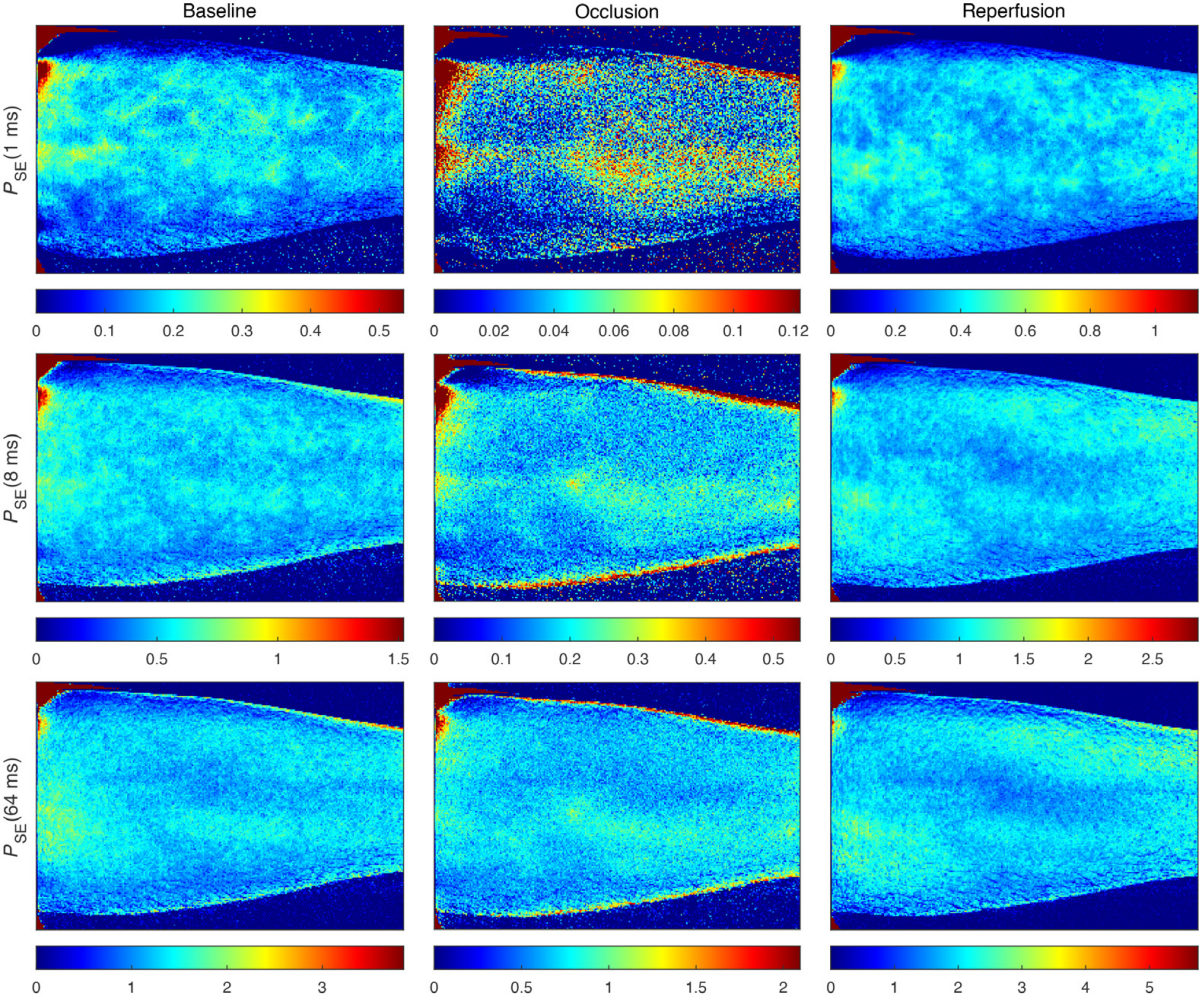
Perfusion estimates based on single-exposure LSCI using the inverse contrast model described in Eq. (6), for exposure times 1, 8, and 64 ms. The color scale in each image was selected for all images to have the same apparent mean, based on the perfusion in the large ROI in Fig. 7. Perfusion values in arbitrary units are presented in the colorbars below each image.

**Fig. 10:**
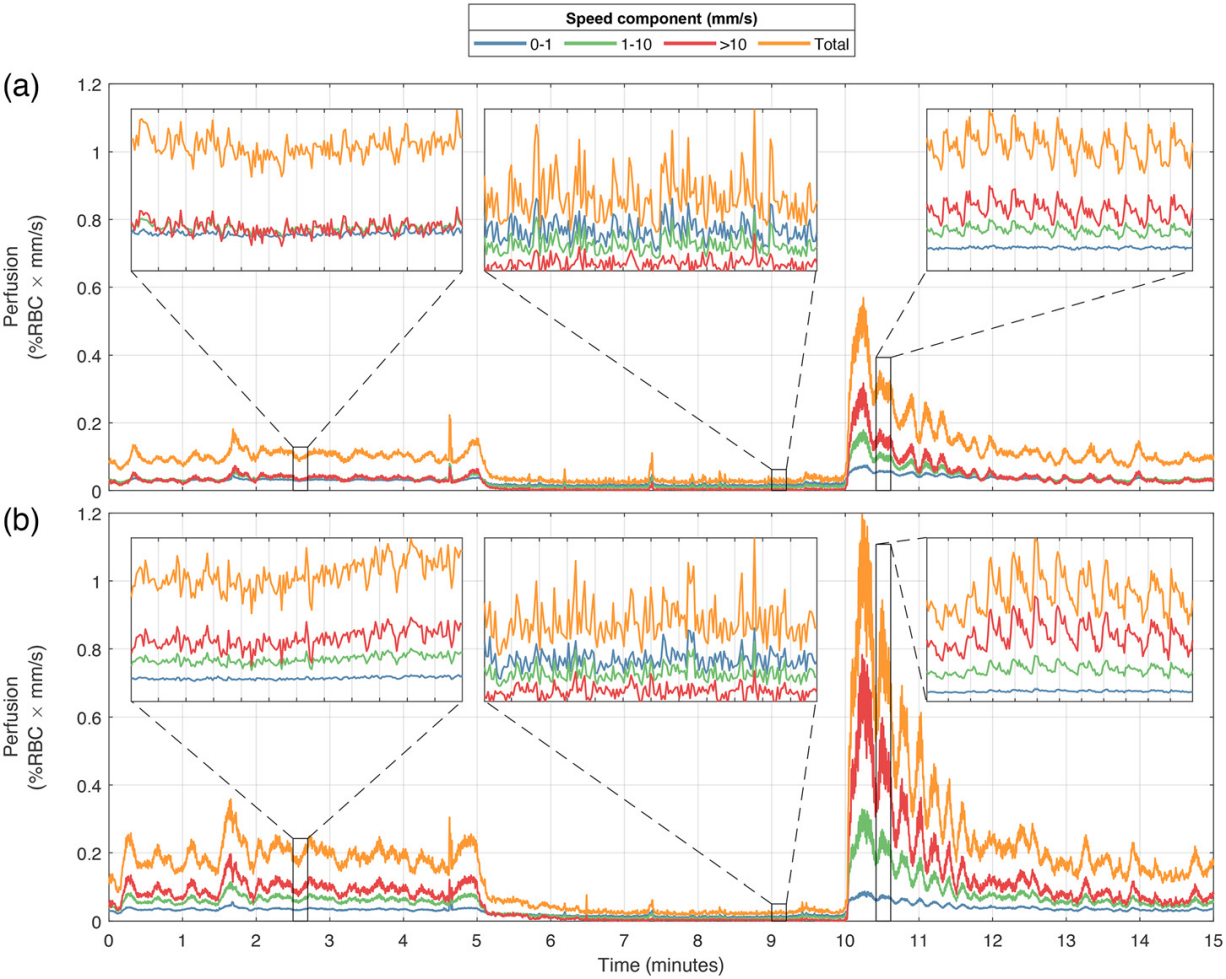
Speed-resolved perfusion in (a) a low-flow ROI and (b) high-flow ROI during the occlusion-release provocation (baseline 0 to 5 min, occlusion 5 to 10 min, reperfusion 10 to 15 min). Care was taken to place the ROIs (see Fig. 7) outside any large vessels visible in the perfusion and intensity images. Zoom plots show 12 s during each of the three phases of the provocation.

**Fig. 11:**
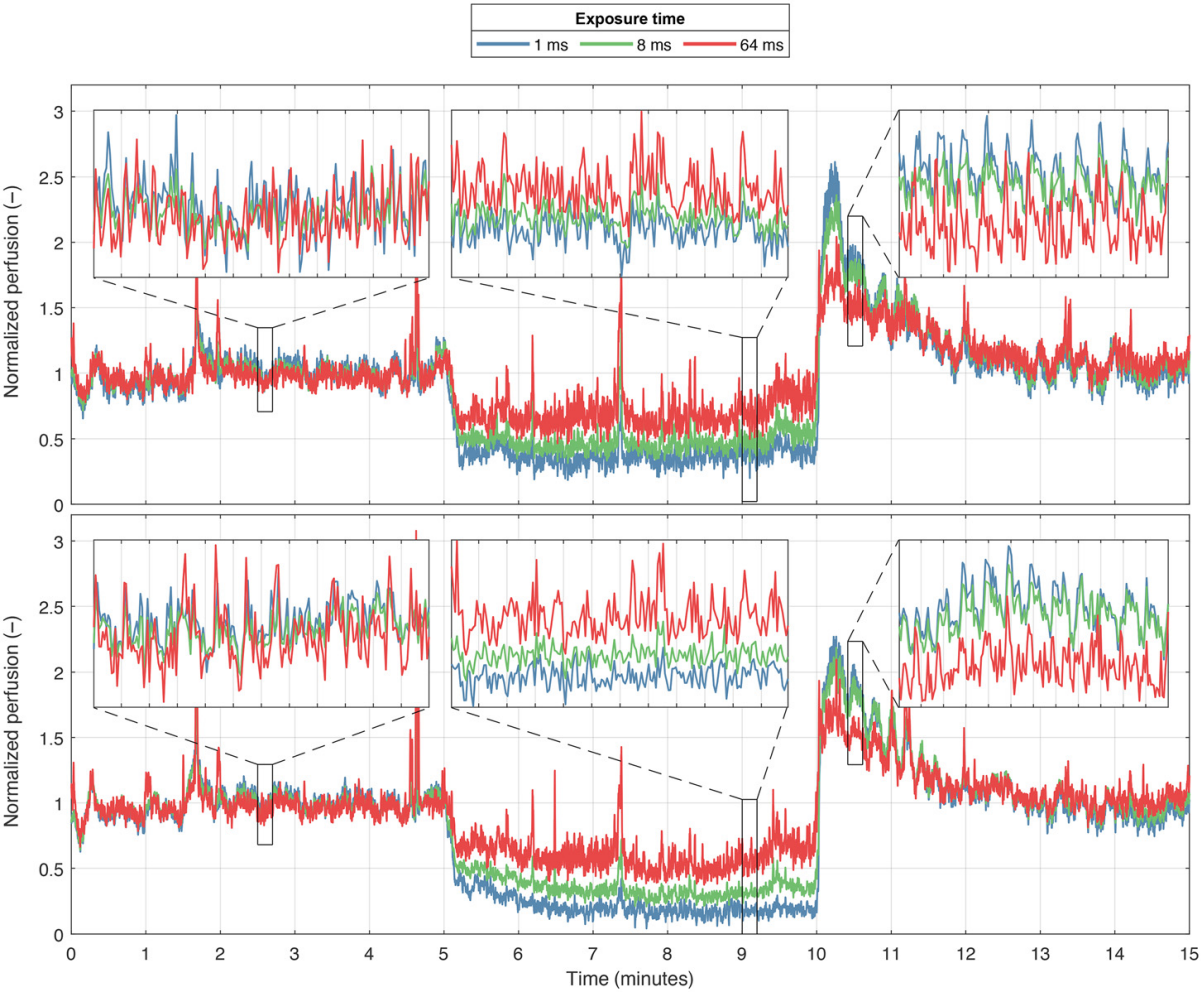
Single-exposure perfusion $P_{\mathrm{SE}}(T)$ at 1, 8, and 64 ms exposure time, for the same ROIs as Fig. 9. The data were baseline-normalized to enable comparison between the different exposure times.

Figure 7 shows an intensity image of the forearm in the occlusion-release experiment and the region-of-interests (ROI) used in the analysis. The large black ROI was used to automatically determine the color scale for the perfusion images in Figs. 8 and 9 and for studying spatial variations in the perfusion. The small ROIs were used to extract time-resolved perfusion for Figs. 10 and 11 and were placed on high-flow (red) and low-flow (blue) regions outside any visible large vessels, to highlight the spatiotemporal heterogeneity of the microcirculation.

Figure 8 presents speed-resolved perfusion images during an arterial occlusion-release provocation of the forearm of a healthy volunteer. The images are time averages over 2 s (32 images), selected at 2:00 min (baseline), 9:00 min (occlusion), and 10:06 min (early after release). The absolute value of perfusion is very different for the different speed components and the three phases of the provocation, making direct comparison difficult when using the same color scale for all images. To visually enhance the similarities and differences in the perfusion measures, the color scale of each image was scaled as three times the corresponding mean value computed in the region marked by the large ROI in Fig. 7 (the factor 3 was chosen empirically). The perfusion scales in the unit %RBC × mm/s are shown in the colorbars below each image. Furthermore, to quantify the heterogeneity of the microcirculation, Table 1 summarizes the coefficient of variation (CV) in the large ROI marked in Fig. 7, for each perfusion image in Fig. 8. The CV increases for the higher speed components, indicating a more spatially heterogeneous distribution of large vessels, assuming that higher speeds are more prominent in larger vessels. This is consistent with the morphology of the microvasculature.^35^ The visible vessels in Fig. 8 are easily observed in the occlusion images for the higher speed components.

**Table 1:** Coefficient of variation (CV) [%] for the perfusion in Fig. 8, computed in the large ROI in Fig. 7.

	Baseline	Occlusion	Reperfusion
0–1 mm/s	13	14	19
1–10 mm/s	22	27	28
>10 mm/s	32	117	36
Total	23	26	31

Figure 9 presents images at the same time-points as in Fig. 8, with perfusion estimated by single-exposure contrast at 1, 8, and 64 ms, according to Eq. (6). Color scales were selected in the same way as described for Fig. 8. The vascular structures observed during occlusion in the ANN perfusion (see Fig. 8; 1 to 10  mm/s, >10  mm/s and total perfusion), are not seen in the corresponding single-exposure images (Fig. 9). The subjective heterogeneity in Fig. 9 is lower than that in Fig. 8, especially for the longer exposure times, but difficult to directly compare due to the increased noise.

Figure 10 presents time- and speed-resolved perfusion of a low-flow region (top) and a high-flow region (bottom), according to the small ROIs shown in Fig. 7. A 0.5-s mean filter was applied to the graphs in the full timescale to suppress the heart-beat variations and increase visibility of long-term changes. The zoomed-in plots show unfiltered data in 12-second sections during baseline, occlusion, and reperfusion. Gridlines in the zoom plots indicate 1-s intervals. Note that for the low-flow ROI, all speed components are of approximately the same magnitude in baseline and at reperfusion, whereas for the high-flow ROI, the high-speed component is dominant at both baseline and at reperfusion. Furthermore, for both the low-flow and the high-flow ROIs, both the 1 to 10  mm/s and the >10  mm/s speed components show heart-synchronous pulsations at reperfusion.

Figure 11 presents single-exposure perfusion [Eq. (6)] in the same two ROIs, at 1, 8, and 64 ms exposure time. The perfusion at each exposure time was normalized to baseline (first 5 min) to allow for comparison between the exposure times.

## 4 Discussion

The method presented in this paper addresses several of the long-standing issues with LSCI and more recently MELSCI; the arbitrary units, limitation to relative measurements, and the inability to distinguish between perfusion from high concentration with low speed and perfusion from low concentration with high speed. The new method allows for direct inter-individual comparisons and the establishment of normative microcirculatory perfusion levels, which potentially increases the clinical usefulness of speckle imaging. The ability to separate slow flow, roughly corresponding to flow in capillaries, from fast flow, roughly corresponding to arterioles and venules, is especially important. This can enable direct analysis of the nutritive capillary flow, which otherwise would likely be masked by the much higher perfusion in the larger vessels.

When evaluating the ANN model, two aspects are especially important to consider. First, the absolute accuracy of the model, and second, the response to changes in the dynamic skin properties. The absolute accuracy for each speed component and the total perfusion is summarized in Fig. 3. We first observe that the restricted model performs very well, indicating that the multi-exposure contrast contains significantly more information about the speed-resolved perfusion than is apparent when only examining the main model. This performance could likely be increased further by extending the range of exposure times, especially to lower values. However, this should be weighed against the increased hardware and processing requirements.

Compared to the restricted model, the main model performs significantly worse. We observed that the estimation error for total perfusion was ∼34%. This shows that the confounding tissue parameters affect the contrast such that the true information about the perfusion is partially lost. This is especially true for the low-speed component (0 to 1 mm/s), which has a nonlinear response to true perfusion when influenced by the effect of tissue parameters and noise. It is possible that this could be addressed by estimating the most important tissue parameters using complementary methods and provide them as input to the model so it can learn to compensate for these effects. This will be an important topic of future research.

It is also important to examine the error values (e.g., MAPE = 34% for total perfusion) in the proper context. As we demonstrate in Fig. 4, the corresponding errors when using single-exposure LSCI is at best 67%, obtained at T=1  ms. The error increases with higher exposure times and is over 100% for T=64  ms. True perfusion is very difficult to estimate from speckle contrast since the contrast is also affected by tissue properties that are independent from perfusion. Conventional LDF perfusion, on the other hand, is affected by the tissue properties in a similar way to speckle contrast. As we have demonstrated in a previous study, contrast from the same exposure times used in this study can be used to estimate LDF perfusion almost perfectly,^2^ but this is not the case for true perfusion.

It is also important to realize that the 34% error does not reflect the uncertainty for relative changes in perfusion for a single individual at a single skin site, but rather the accuracy across the whole dataset with very wide distributions of skin properties, as seen in Fig. 1. To better describe how the model behaves in a single individual, the analysis in Fig. 5 is more relevant, where we see that the model has a linear response to both blood concentration and mean blood speed. In other words, given a single individual, the absolute accuracy in perfusion is determined by the static skin properties, but the model responds linearly to changes in perfusion, for example during provocations. This also indicates that perfusion from tissues with similar properties can likely be compared with a much smaller variation than 34%. For example, measurements on the forearm in different individuals would likely fall in the same region in the space of tissue properties, thus resulting in comparable perfusion values between individuals. Conversely, comparing perfusion in the forearm and palm might give higher variations even in a single individual, due to the differences in tissue properties.

When examining Fig. 5, we observe some deviations in the estimated $P_{\mathrm{ANN},\mathrm{tot}}$ from the ideal line. $P_{\mathrm{ANN},\mathrm{tot}}$ slightly overestimates perfusion at high average speeds, and begins to deviate from the true value $P_{\text{true},\mathrm{tot}}$ at low blood tissue fractions (<0.2%), where it has a less linear response than the single-exposure perfusion estimates $P_{\mathrm{SE}}(1\;\mathrm{ms})$ and $P_{\mathrm{SE}}(8\;\mathrm{ms})$. The combined mean percentage deviation for Figs. 5(a) and 5(b) is $P_{\mathrm{ANN},\mathrm{tot}}$: 8%, $P_{\mathrm{SE}}(1\;\mathrm{ms})$: 14%, $P_{\mathrm{SE}}(8\;\mathrm{ms})$: 17%, and $P_{\mathrm{SE}}(64\;\mathrm{ms})$: 44%. Thus, when considering the combination of linearity to both parameters, $P_{\mathrm{ANN},\mathrm{tot}}$ is overall the stronger model.

Figure 5 also demonstrates that the choice of exposure time for single-exposure LSCI has a large impact on the accuracy of the perfusion estimates relative to speed changes. Lower exposure time results in a more linear response to changes in the speed, whereas an exposure time of 8 ms is almost perfectly linear relative to changes in blood tissue fraction. It has been suggested that 5 ms is the optimal exposure time for LSCI,^36^ but this will likely depend on the application.

Figure 6 summarizes the largest contributing factors to the error in estimated perfusion. For different values of scattering and vessel diameter, we observe an almost unnoticeable change in true perfusion, whereas all perfusion estimates are significantly affected. The small change in true perfusion is due to minor changes in the sampling volume. Changes in the epidermis thickness have a small effect on the true perfusion, and slightly higher effect on the perfusion estimates, especially for thin epidermis layers. The effect is smaller on $P_{\mathrm{ANN}}$ than on all single-exposure estimates for changes in scattering, vessel diameter, and epidermis thickness. We also performed this analysis for changes in oxygen saturation and epidermis absorption coefficient (representing melanin concentration) and found expectedly low changes in both $P_{\mathrm{ANN}}$ and $P_{\mathrm{SE}}$ of <0.5% and 2% across the whole tested ranges, $S_{\mathrm{oxy}}\in [0,100]\%$, $\mu_{a,\mathrm{epi}}\in [0.01,3.5]\;{\mathrm{mm}}^{-1}$, respectively. However, the effect of those parameters is likely higher if a laser with a shorter wavelength than 780 nm is used, where the absorption is generally higher both for melanin and hemoglobin, and generally differs more between oxygenized and reduced hemoglobin. In summary, the analysis of Fig. 6 demonstrates that some skin properties, especially scattering and vessel diameter, affect the speckle contrast in a way that cannot be decoupled from changes in blood concentration or speed, thus affecting the estimated perfusion. The magnitude of the effects can be decreased by limiting the range of parameter values in the training data, but this might also have the opposite effect if the distributions are too narrow to accurately cover all tissues in real data. The influence of tissue properties on the absolute accuracy of the perfusion estimate will be an important topic of research for future studies. Since these properties cannot be directly measured using MELSCI, solutions will likely require integrating additional modalities into the imaging system.

Figure 8 presents speed-resolved perfusion images from the arterial occlusion-release provocation. The first observation is that the subjective image quality of these perfusion estimates is higher than in the single-exposure perfusion estimates in Fig. 9, both in terms of less noise and in providing visible vessel structures. It is worth noting that the single-exposure estimates are based on exactly the same 64 ms of data as the ANN perfusion estimate. For example, the 8 ms single-exposure contrast is the average of 8 consecutive contrast images during the 64 ms. See Ref. 18 for the details of the contrast algorithm. While the absolute value of the perfusion on the large vessels should be interpreted carefully since the ANN model is trained to estimate microcirculatory perfusion, the fact that they are clearly visible is important, as this means that further analysis can be performed on skin regions that do not contain the large vessels. Since most of the large vessels are not visible in the single-exposure images, with these methods, there is a risk of analyzing tissue that is not representative of the microcirculation.

We can observe how the perfusion becomes more concentrated to high-perfusion sites for higher speed components. This indicates an increased sensitivity to perfusion in larger vessels, which are expected to be more heterogeneously distributed and have higher flow speeds. However, the anatomical interpretation of these high-flow spots deserves further studies. To quantify the heterogeneity, we computed the coefficient of variation (CV) for each image in Fig. 8, which is summarized in Table 1. Here we observe that the CV increases toward higher speed components, corresponding to the more heterogeneous perfusion. This effect is especially noticeable in the images during the occlusion, where the perfusion in the 0 to 1  mm/s interval is more homogeneous, whereas the >10  mm/s interval has perfusion mostly concentrated in the large vessels, with low perfusion in between the large vessels. The perfusion in the large vessels during the occlusion is possibly due to Brownian motion in the blood, although these perfusion values should be interpreted with care since the training dataset did not include this morphology.

In Fig. 10, we present the speed-resolved perfusion in two ROIs, manually selected on a low-flow and a high-flow perfusion site by inspecting the images in Fig. 8. This demonstrates the two extremes of the heterogeneous perfusion, with an ∼2 times higher reperfusion peak for total perfusion in the high-flow ROI compared to the low-flow ROI. The zoomed-in plots in Fig. 10 show the different speed components in more detail. Note that in both ROIs during the occlusion, the 0 to 1  mm/s perfusion is the highest of the three speed components, corresponding to a low-speed Brownian motion, and the >10  mm/s perfusion is the lowest. For the high-flow ROI, this is the opposite order compared to baseline. In the reperfusion phase, the 0 to 1 mm/s component is the lowest and the >10  mm/s component is the highest. This behavior is expected and demonstrates multiple large shifts in the speed distribution during the experiment. The heart-beat signal is also clearly visible during the reperfusion for the two highest speed components, and a strong flowmotion at ∼0.075  Hz (13 s period) can be seen especially in the high-flow ROI, indicating high myogenic activity.^37^^,^^38^ If we inspect the amplitudes of cardiac-related pulses in the reperfusion phase, we find that it is ∼4 times higher in the high-flow ROI compared with the low-flow ROI. This further indicates that the high-flow ROI contains larger vessels where cardiac pulses are more visible.

For comparison, in Fig. 11 we present the same ROIs analyzed with the single-exposure perfusion model [Eq. (6)]. The single-exposure perfusion from different exposure times is naturally similar since the data were normalized to baseline to allow comparison, but we also observe that the differences between the low-flow and high-flow region are smaller than in the speed-resolved data in Fig. 10. It is also apparent that the noise level is higher compared to the ANN model. This is expected since the ANN is specifically trained to account for the measurement noise present in the speckle contrast, and the single-exposure model is not.

There are a few limitations to this work, and some disadvantages compared to conventional single-exposure LSCI. First, our model is specifically trained on a dataset mimicking skin tissue. While it would be possible to change the model to target other tissues, this was not a part of this work. Second, the combination of the complex tissue model and machine learning makes it difficult to investigate the reason our method works or the cases when it fails. The benefits of our model should be weighed against the higher transparency inherent in a model such as Eq. (6), or other MELSCI models (e.g., Ref. 7). The complex model, in combination with the custom MELSCI system, also makes replication of this study quite difficult. Another limitation of the current study is that we did not perform validation measurements in a controlled flow phantom. Given the complex tissue morphology targeted by our model, such validation setups are likely difficult to achieve. However, the main components of the presented methodology have been independently validated in previous studies.^2^^,^^14^

Overall, the results presented in this paper illustrate possible analyses enabled by speed-resolved perfusion. A similar principle has been useful for analysis in a pointwise measuring system, where a reduced low-flow speed-component, interpreted as reduced nutritive capillary flow, could be observed in patients with type 2 diabetes compared to healthy controls.^15^^,^^39^ For speed-resolved perfusion imaging, it is reasonable to assume that the added spatial information can remove the uncertainty present in single-point measurements due to the spatial heterogeneity in the microcirculation. Furthermore, speed-resolved perfusion imaging potentially enables applications where both the spatial heterogeneity and the separation of low-speed from high-speed vasculature are clinically relevant, such as in sepsis.^40^^,^^41^

## 5 Conclusion

We have presented a method for assessing speed-resolved perfusion in an imaging modality. The distinction between physiologically relevant speed components and the absolute units %RBC × mm/s has the potential to greatly improve the physiological interpretation and the clinical impact of measurements using MELSCI.

## Code, Data, and Materials Availability

Please contact the authors regarding availability of the code and data used in this study.
